# MicroRNAs in the oriental fruit fly, *Bactrocera dorsalis*: extending Drosophilid miRNA conservation to the Tephritidae

**DOI:** 10.1186/s12864-015-1835-3

**Published:** 2015-10-05

**Authors:** Bernarda Calla, Scott M. Geib

**Affiliations:** Tropical Crop and Commodity Protection Research Unit, USDA-ARS Pacific Basin Agricultural Research Center, Hilo, HI 96720 USA

**Keywords:** Transcriptome, Hairpin, mirDeep, Oriental fruit fly, miRNA clusters

## Abstract

**Background:**

The oriental fruit fly, *Bactrocera dorsalis,* is an important plant pest species in the family Tephritidae. It is a phytophagous species with broad host range, and while not established in the mainland United States, is a species of great concern for introduction. Despite the vast amount of information available from the closely related model organism *Drosophila melanogaster*, information at the genome and transcriptome level is still very limited for this species. Small RNAs act as regulatory molecules capable of determining transcript levels in the cells. The most studied small RNAs are micro RNAs, which may impact as much as 30 % of all protein coding genes in animals.

**Results:**

We have sequenced small RNAs (sRNAs) from the Tephritid fruit fly, *B. dorsalis* (oriental fruit fly), specifically sRNAs corresponding to the 17 to 28 nucleotides long fraction of total RNA. Sequencing yielded more than 16 million reads in total. Seventy five miRNAs orthologous to known miRNAs were identified, as well as five additional novel miRNAs that might be specific to the genera, or to the Tephritid family. We constructed a gene expression profile for the identified miRNAs, and used comparative analysis with *D. melanogaster* to support our expression data. In addition, several miRNA clusters were identified in the genome that show conservancy with *D. melanogaster*. Potential targets for the identified miRNAs were also searched.

**Conclusions:**

The data presented here adds to our growing pool of information concerning the genome structure and characteristics of true fruit flies. It provides a basis for comparative studies with other Dipteran and within Tephritid species, and can be used for applied research such as in the development of new control strategies based on gene silencing and transgenesis.

**Electronic supplementary material:**

The online version of this article (doi:10.1186/s12864-015-1835-3) contains supplementary material, which is available to authorized users.

## Background

True fruit flies (Diptera: Tephritidae), constitute a family of mostly phytophagous species, many of which are considered to be serious pests of numerous plants. Within this family, species in the highly invasive genera *Anastrepha*, *Bactrocera*, *Ceratitis* and *Rhagoletis* are of worldwide economic importance, restricted by quarantine listings in Europe [1], and subject to constant eradication and establishment prevention in the United States. Research on this family of flies has been heavily concentrated on field strategies and quarantine methods [2]. Despite the vast amount of information available from the closely related model organism *Drosophila melanogaster*, information at the genome and transcriptome level is still very limited in true fruit flies.

The regulation of gene expression in the cell is driven by fine-tuned molecular mechanisms that respond to developmental and environmental cues. Playing an important role in this system are the small RNAs (sRNA) which act as regulatory molecules capable of determining target mRNA expression levels [3, 4]. Several types of sRNAs have been documented to date. Among the most studied are micro RNAs (miRNAs) and small interfering RNAs (siRNAs); both types present in most species. Classes of sRNAs and their biogenesis pathways have been extensively described in the literature for many model species [3–6]. MiRNAs originate in genomic loci and are very often expressed in a tissue-specific manner. A large proportion, probably more than 30 %, of all protein coding genes of animals may be regulated by miRNAs.

We have sequenced sRNAs from the Tephritid fruit fly, *Bactrocera dorsalis* (Oriental fruit fly), specifically sRNAs corresponding to the 17 to 28 nucleotides long fraction of total RNA, looking at variation in composition and expression between different developmental stages, and between male and female sexes in the pupa stage. We were able to identify several miRNAs orthologous to known miRNAs and additional novel miRNAs that might be specific to the genera or to the Tephritid family. We constructed a profile of gene expression for the identified miRNAs, and used comparative analysis with *D. melanogaster* to support our expression data, identify conserved miRNA clusters in the genome, and mine for potential transcript targets for this miRNAs. The data presented here adds to the biological information concerning the genome structure and characteristics of true fruit flies. It provides a basis for comparative studies in other Dipteran species, and can be used for applied research such as in the development of new control strategies based on gene silencing and transgenesis.

## Methods

### Fly sample collection

A white pupal translocated strain (DTWP) was used, in which female pupae are white and male pupae are brown, allowing for separation of sex in the pupal stage [7]. Flies were grown in liquid diet [8], (200 ml diet for approximately 3400 eggs) as previously described [9].

Three biological replications were carried out for sample collection, RNA extraction and sequencing. For each replicate, eggs were allowed to develop and samples collected at the following times and developmental stages: embryos (12 mg at 0–1 h after oviposition), young larvae (approximately 20 mg at 0–12 h after egg hatch), early male (brown) pupae (0–24 h after pre-pupal formation), and early female (white) pupae (0–24 h after pre-pupal formation). For embryos and larvae, samples were sieve washed and then blot dried. All samples were collected in 1.5 ml microcentrifuge tubes and flash frozen in liquid nitrogen immediately after collection. Samples were then stored at −80 °C until processed. For fertilized ovary collection, 7 day old females and males were left in a cage to mate. Thirty seven mating pairs were separated in cups and left for at least 90 min to make sure that the females were fertilized. Non-mating flies were removed and mating pairs were left until the next day. Mated females were sedated by exposing them to 4 °C for 10 min, and the ovaries were dissected. Ovaries from ten female flies were collected per replication in 1.5 ml microcentrifuge tubes and flash frozen in liquid nitrogen. Samples were preserved at −80 °C until RNA extraction.

### RNA extraction

RNA from each of the collected samples was extracted utilizing NucleoSpin® miRNA kit (Macherey-Nagel, Duren, Germany) following manufacturer’s protocol and recommendations. Initial tissue lysis was performed by grinding the frozen tissue in 1.5 ml tubes with plastic micro pestles, followed by addition of 300ul of lysis buffer. The NucleoSpin miRNA kit allows for separation of small RNA and large RNA fractions in silica membrane columns by differential ethanol concentrations. After purification, the quality and quantity of both the small and large RNA fractions for each sample was determined using a Quibit 2.0 fluorometer (Life Technologies, Carlsbad, California), and an Agilent 2100 Bioanalyzer with anAgilent small RNA kit (Ambion, Santa Clara, CA, USA).

### Library preparation and sequencing

To prepare small RNA sequencing libraries, the Ion Total RNA-seq kit v2 for small RNA libraries was used following manufacturer protocols with some modifications. The small RNA fraction of each sample was ligated to adapters and reverse transcribed to cDNA. The cDNA was purified, size selected and each sample was differentially barcoded before amplification to allow subsequent sample identification. Amplified cDNA was checked for quality and size distribution using an Agilent 2100 Bioanalyzer with the High Sensitivity DNA assay kit. To further size select the cDNA, a BluePippin® (Sage Science, Beverly, MA) was used with a 3 % agarose gel cassette, enabling the enrichment of cDNAs between approximately 92 and 118 bp, (corresponding to small RNAs of 18–27 nucleotides length with the addition of sequencing adaptors and barcodes). Afterward, equimolar amounts of samples (identifiable by differential barcodes) were pooled, and the pooled library was quantified using a KAPA library quantification kit (KAPA Biosystems Woburn, MA) to assess the optimal amount for emulsion PCR. Emulsion PCR was performed on an Ion One Touch 2 System and the amplified beads were subjected to sequencing with the Ion Personal Genome Machine using an Ion PGM Sequencing 400 kit and Ion 318 Chip v2. To maximize reads per sample, sequencing runs were performed using 120 flows per run, followed by repeated sequencing of the library a total of 6 times across two initializations of the PGM instrument with the 400 kit. Post sequencing base calling, adapter trimming, and demultiplexing was performed using the Torrent Suite Software using default parameters for small RNA sequencing, and exported as fastq files.

### Identification and classifications of small RNA sequences

The resulting 14 fastq files corresponding to a replicate sample from each life stage were entered separately in the mapper.pl module of the mirDeep2 package [10, 11], using a config.txt file to track the original sample in the final results. The mapper.pl module discarded reads smaller than 17 nt (option -l 17), collapsed identical reads and performed counting. Additionally, reads were mapped to the *B. dorsalis* genome (GeneBank Accession # JFBF00000000.1) using bowtie [12] with the following stringency parameters: 0 mismatches allowed in the seed, seed length of 18 nt, up to two mismatches after the seed, discarding reads mapping more than 5 times to the genome, and reporting only the best alignment for each read. The pipeline was designed to predict high-confidence miRNAs, and while discarding mature sequences that map to too many loci compromised detection, it also prevented false positive predictions. The collapsed reads obtained from the mapper.pl module were input into the miRdeep2 core module (miRdeep2.pl) with no reference miRNAs from a closely related species supplied, in this manner all the potential miRNAs and precursors from the data could be obtained. In the miRDeep2 module, read mappings were used to excise putative miRNA precursors according to stack height and all sequenced reads were aligned to these potential precursors. Additionally, the secondary hairpin structure and its stability were predicted for the excised precursors utilizing RNAfold [13–15] and Ranfold [16], and a score was assigned to each precursor. Scores are used to select precursors with highest probability of being genuine (the program kept precursors with scores higher than −50). To find other non-miRNA small RNAs, mapped reads (from mapper.pl module output) were scanned against the covariance models of the Rfam 11.0 release [17], using Infernal 1.1.1 cmscan [18].

The list of potential mature miRNAs and hairpin precursors obtained from the miRDeep2 core module were subjected to nucleotide BLAST search against the Sanger miRBase mature.fa and hairpin.fa database respectively (database release 21, http://www.mirbase.org) [19–21], this was done with the aim of discriminating known miRNAs and iso-miRNAs from potential novel miRNAs. Nucleotide BLAST for mature sequences was performed utilizing the blastn program with the blastn-short task default parameters, except for word-size which was changed from the default of 7 to 16; in this manner, only nearly identical sequences were identified. The resulting list was further filtered to keep only perfect and near-perfect matches to known miRNAS (near perfect defined as having one mismatch or up to a 2 nucleotide length difference between the query and subject sequences). BLAST for hairpin precursors was run with standard blastn with a word size 16 and cut-off e-value 1e-7, and the result was filtered to remove instances where the potential precursor was more than 8 bases longer than the aligned stretch.

### Target identification

To identify potential targets, the 3’UTR region was obtained from predicted gene annotations in the *B. dorsalis* genome assembly (GeneBank Accession # JFBF00000000.1), annotations were downloaded from USDA i5k web portal at https://i5k.nal.usda.gov/content/data-downloads. The 3’UTRs were compared against the identified miRNAs using miRANDA [22, 23]. A stringent threshold was applied for a conservative approach (pairing score: >155, energy score: <−7, gap opening penalty: −8 and Gap extension penalty: −8).

### Differential expression of miRNAs

To assess the expression changes for the identified miRNAS across the life stages tested, the raw counts for the identified miRNAs obtained from the MirDeep2 quantifier.pl module were input into the EdgeR [24, 25], utilizing the pairwise exact-test modality to test each of the 11 possible comparisons on TMM (trimmed means of M-values) normalized counts. Correlations between miRNA expression in *B. dorsalis* and *D. melanogaster* were computed utilizing Spearman correlation (ρ). The method was chosen because we do not expect a perfect linear relationship between miRNA levels in both species due to developmental timing differences, Spearman correlation calculates coefficients based on rank. All figure generation and statistics were performed in R.

## Results and discussion

### Sequencing results

After quality filtering, the six sequencing runs of the pooled libraries yielded more than 16 million reads in total. While most libraries had approximately one million reads or more, the “ovary replication 1” library had less than 100,000 usable reads, representing an outlier in our dataset. The “female pupa replication 2” sample failed to produce the necessary amount of cDNA in three attempts, and thus was not sequenced. Because of the stringent size-selection used in cDNA previous to sequencing of the libraries, only a small portion of the total reads (4.7 %) were removed at the 17 nt cutoff. The remaining reads (with more than 17 nt) were collapsed into identical reads in a library per library basis resulting in libraries with as low as 18 thousand unique reads (ovary replication 1), to libraries with more than 500 thousand unique reads (ovary replication 3). About 23 % of the unique reads across libraries mapped to at least one location in the *B. dorsalis* genome (Table 1).

**Table 1:** Summary of sequencing results. Number of sequenced, processed and aligned reads

Library	Total number of sequenced reads	Total usable reads equal to or longer than 17 nt	Total reads mapped	Number of unique sequences	Number of unique sequences mapping to at least one and up to 4 locations in the genome	Unique sequences matching an Rfam model (non-coding RNA)
Eggs (rep1)	661,329	649,691	153,904	168,959	46,142 (27.31 %)	3,471 (2.05 %)
Eggs (rep2)	1,783,633	1,755,385	391,420	415,781	114,655 (27.58 %)	6,723 (1.61 %)
Eggs (rep3)	1,511,629	1,495,468	274,923	339,551	97,218 (28.63 %)	5,117 (1.51 %)
Larvae (rep1)	990,551	901,206	181,715	199,654	55,738 (27.92 %	5,156 (2.58 %)
Larvae (rep2)	1,382,185	1,286,889	181,896	303,152	71,141 (23.47 %)	4,645 (1.53 %)
Larvae (rep3)	730,458	697,460	102,637	172,540	41,204 (23.88 %)	3,452 (2.00 %)
Male Pupae (rep1)	1,659,261	1,502,737	237,960	258,875	39,084 (15.10 %)	4,298 (1.66 %)
Male Pupae (rep2)	1,175,996	1,070,491	112,938	164,853	23,601 (14.32 %)	2,988 (1.81 %)
Male Pupae (rep3)	1,253,417	1,206,485	178,091	176,704	25,543 (14.46 %)	3,472 (1.96 %)
Female Pupae (rep1)	1,086,136	1,017,418	115,176	174,118	19,855 (11.40 %)	2,501 (1.43 %)
Female Pupae (rep2)	--	--	--	--	--	--
Female Pupae (rep3)	1,169,052	1,096,264	169,583	153,575	23,841 (15.52 %)	3,239 (2.1 %)
Ovaries (rep1)	79,391	73,001	10,453	18,251	3,270 (17.92 %)	598 (3.27 %)
Ovaries (rep2)	1,154,349	1,134,024	229,095	398,853	111,069 (27.85 %	6,114 (1.53 %)
Ovaries (rep3)	1,740,634	1,710,835	358,303	523,507	145,910 (27.87 %)	8,025 (1.53 %)
TOTAL	16,378,021	15,597,354			818,271	59,799
					(23.60 % of unique reads)	(27 % of unique reads)

Mapped reads were searched for homologs of structural RNA sequences in the Rfam database to assess the non-coding RNA composition of the dataset. Over 1 million reads matched at least one type of RNA, the most abundant being tRNAs (64 %) and miRNAs (22.5 %). The most frequent RNA types (number of unique sequences in a family type) were tRNAs (16 % of all unique sequences having a match in Rfam), miscellaneous cis-regulatory elements (14 %), miRNAs (14 %), and snoRNAs (11 %) (Table 2). A relatively high number of tmRNAs (transfer-messenger RNA) and CRISPR-like sequences were found (8.3 and 7.3 % of unique sequences per library respectively), indicating the presence of bacteria in the sequenced tissues, however, the total amount of reads in these categories was relatively low: 3.3 and 2.9 % respectively. The full Rfam classification including frequency, abundance and diversity is detailed in Additional file 1: Table S1.

**Table 2:** Rfam classification. Number of unique sequences classified into each of the 12 main types of non-coding RNAs

	Eggs			Larvae			Male pupae			Female pupae		Ovaries		
Type	E01	E02	E03	L01	L02	L03	BP1	BP2	BP3	WP1	WP3	OV1	OV2	OV3
tRNA	788	927	317	1071	504	614	998	730	928	659	867	143	592	839
miRNA	433	901	515	653	782	542	912	551	599	588	660	280	604	748
sno-RNA	395	1018	918	535	577	355	291	186	176	157	189	23	928	1165
tmRNA	416	347	199	773	299	438	635	615	759	328	612	51	270	427
sRNA	248	593	496	321	383	221	238	137	163	108	146	17	606	778
CRISPR	209	584	524	272	372	198	224	136	143	115	128	20	652	815
rRNA	122	283	225	170	248	151	230	174	191	141	162	23	238	381
HACA-box	94	201	187	132	126	112	68	47	45	45	50	6	205	270
splicing	63	87	80	318	208	157	34	27	31	35	35	2	152	197
riboswitch	33	124	106	48	74	40	52	27	33	24	19	3	128	146
misc. cis-reg	507	1212	1157	581	756	424	403	250	276	198	264	18	1250	1619
others	163	446	393	282	316	200	213	108	128	103	107	12	489	640
TOTAL	3471	6723	5117	5156	4645	3452	4298	2988	3472	2501	3239	598	6114	8025

### Identification of known and novel miRNA in the dataset

MirDeep2 identified 149 potential miRNAs among the mapped reads in the pooled library, and the software assigned a provisional identification (provisional ID) to each of them. In five instances, two or three different provisional IDs were assigned to the exact same sequence because they aligned to more than one precursor excised from different genome scaffolds or from different regions in the same scaffold. The output was further filtered for miRNAs with a miRDeep2 score below or equal to 2, as a visual examination of the predicted hairpin structures and “stacks” built with the reads revealed that these hairpins had low signal to noise ratio (number of miRNA vs miRNA* and other reads in the region), and unusual secondary structures that did not resemble typical metazoan miRNA precursors [26, 27]. The remaining potential miRNA hairpins were screened to verify if they were contained within or overlapped predicted gene exons in the *B. dorsalis* genome. While the majority of the hairpin precursors were located outside of gene-coding regions and in introns, 13 of them were found either contained within an exon (10), contained a full exon (2), or overlapped an exon start (1). Whereas miRNAs are traditionally found in non-gene-coding-regions, we did not exclude these sequences from further analyses, as some more recently identified miRNAs in *D. melanogaster* were found within exonic regions, including 3’UTRs and coding sequences (CDSs) [28]. In addition, because the gene set being used is largely computationally predicted, some errors in gene structure may be present.

**Fig. 1:**
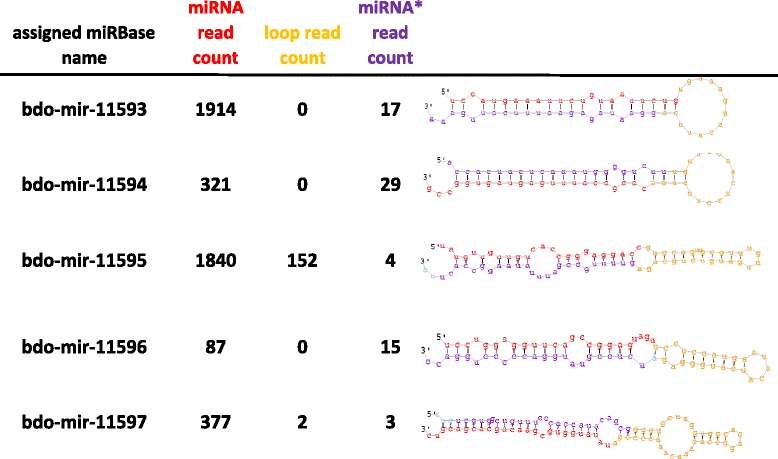
Sequencing of the small RNA fraction of *Bactrocera dorsalis* at five different developmental stages identified five potential novel miRNAS. The precursor hairpin structures and counts of miRNA and miRNA* are shown. In the hairpin, the mature miRNA is colored *red*, the loop sequence is colored *yellow*, and the miRNA* sequence is colored *purple*

The described filtering yielded 109 potential miRNAs, which were used for BLAST homology search against the miRBase mature miRNAs (mature.fa) and precursors (hairpin.fa) datasets for further validation. A total of 74 of the potential miRNAs had homology to either a hairpin precursor, a mature miRNA, or both in miRBase (Additional file 2: Table S2). For the remaining 35 sequences that did not match known sequences in the miRBase database, additional information was collected to prove or disprove their authenticity as novel miRNAs. For that purpose, and given that the fold-back precursors for these sequences were already detected in the genome, expression levels were used as a secondary supporting criterion [29]. One of these reads was present in all libraries, while one other miRNA was missing in only one of the larvae replications, but was consistently low in the other two replications for this stage, indicating possible low tissue or developmental stage expression. These two sequences were selected as putative novel miRNAs. Additionally, seven other sequences were present in all except male and female pupae libraries. Three of these seven had low read count and/or there were no reads detected for their corresponding miRNA*. For one other, visual examination of the hairpin revealed unusual secondary structure. These four sequences were eliminated, leaving three additional potential novel miRNAs for a total of five. The remaining 29 sequences were present in only one replicate of a single developmental stage, and were disregarded. The predominant sequence in all of the five selected cases was the miRNA relative to the miRNA*, and none of these were found in or overlapping exonic regions, further supporting these sequences as true novel miRNAs (Table 3 and Fig. 1).

**Table 3:** Putative Novel miRNAs identified in *Bactrocera* dorsalis (orthologous to these miRNA sequences were not previously reported in other species). See also Fig. 1

Genomic location	miRBase assigned ID	Total read count	Consensus mature sequence	Eggs	Larvae	Male pupae	Female pupae	Ovaries
scaffold00003_210	bdo-mir-11593	1931	uccaugaaauucuguaauucug	yes	yes	yes	yes	yes
scaffold00003_276	bdo-mir-11594	350	cacgccauuugaguaguggccg	yes	yes	yes	yes	yes
scaffold00155_6328	bdo-mir-11595	1996	uauguuguugucaccgggaggacc	yes	yes	no	no	yes
scaffold01114_17715	bdo-mir-11596	102	uccuggagguucagccggaguagu	yes	yes	no	no	yes
scaffold01468_19863	bdo-mir-11597	382	uauauggugcgaacagcacgacguc	yes	yes	no	no	yes

In animals, miRNAs regulate transcript abundance by complimentary base pairing to the 3’ UTR of the target RNA, with some exceptions [5]. Potential targets for the 109 identified miRNAs were screened within the set of 3’UTR regions extracted from our most current *B. dorsalis* genome assembly (NCBI Assembly# ASM78921v2) utilizing the miRANDA software. The number of targets identified per each miRNA ranged between 5 and 469, even with the stringent parameters used for target detection, giving a total of 6506 predicted miRNA-target pairs, where many mRNA were targeted by multiple miRNAs. (Additional file 3: Table S3).

### Differential expression of *B. dorsalis* miRNAs

The relative differential abundance of miRNAs between life stages was calculated using the negative binomial distribution with edgeR [25, 30]. In total, 47 of the 80 high confidence microRNAs identified showed differential expression (FDR corrected p-value <0.05) in at least one of the 10 possible pairwise comparisons between life stages. These significantly differentially regulated miRNAs included all 11 novel miRNA sequences. The comparison between female and male pupae yielded no differentially regulated miRNAs, and the most pronounced changes were observed between ovaries and pupae, and between eggs and pupae (Additional file 4: Table S4).

**Fig. 2:**
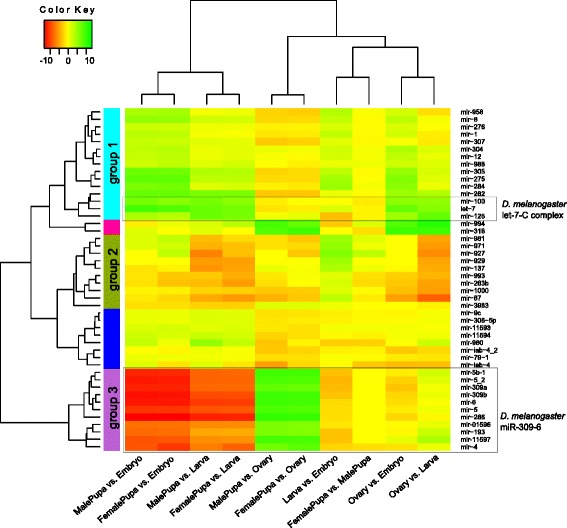
Hierarchical clustering of statistically differentially regulated *B. dorsalis* miRNAs. The clustering allowed for the classification of groups of miRNAs with increased expression during the developmental progression from embryo to larva, embryo to pupa and embryo to female reproductive tract (*group 1*). A group of potential larva-specific miRNAs (*group 2*), and a group of miRNA with very low expression in pupa (*group 3*). The values used for clustering were in the form of log2 ratios to give equal weight to up-regulated miRNAs and down-regulated miRNAs (e.g. MalePupa/Embryo means the log2 of the ratio of MalePupa counts per million over Embryo counts per million). Enclosed in rectangles are genes with orthology to two of the known *D. melanogaster* miRNA clusters

Hierarchical clustering was used to identify patterns of expression across developmental stages. Some well-defined groups of miRNAs could be detected in a heatmap representation of the clustering (Fig. 2). The first group (Fig. 2, group 1) comprised miRNAs with increased expression during the developmental progression from embryo to larva, embryo to pupa and embryo to female reproductive tract. A subgroup of these showed little or no change between larvae to pupa and reduced expression in the transition between pupa to ovary, while a second subgroup had strong up-regulation in pupa compared to larva and little or no change between ovary and pupa. In this last subgroup, the three miRNAs with homology to the *D. melanogaster* polycistronic locus *let-7-Complex* (*let-7-C*) (i.e. mir-100, mir-125, and let-7) were present. This cluster of miRNAs is known to be involved in the timing of cellular development in *D. melanogaster* [31]. In particular, let-7 is known to promote the transition from larva to adult, and is required for remodeling the neuromusculature during metamorphosis. In conformance with our *B. dorsalis* data, let-7 in *D. melanogaster* is also expressed mainly at the pupal stage [32, 33]. A second group in the cluster (Fig. 2, group 2) shows potential larva-specific miRNAs. These miRNAs increased in abundance in larva compared to embryo, but were markedly reduced in female and male pupa compared to larva. Although this group of miRNAs is mainly composed of miRNAs with homology to known miRNAs in *D. melanogaster* or other species, they have been found mainly in genome-wide screenings and their specific functions are unknown [34, 35]. Finally, another well-defined cluster was formed, showing 11 miRNAs highly reduced in abundance in pupa compared to embryo and larva. These miRNAs showed increased abundance in ovary compared to pupa and all the other comparisons between larva, embryo and ovary showed little or no change in expression, indicating that these miRNAs are depleted in pupae and/or highly and equally expressed in embryo and ovary. Six out of the 11 novel *B. dorsalis* miRNAs belong to this group, as did miRNAs belonging to the *D. melanogaster* miR-309-6 cluster (i.e. mir-4, mir-5 and mir-286), (Fig. 2, group 3).

**Fig. 3:**
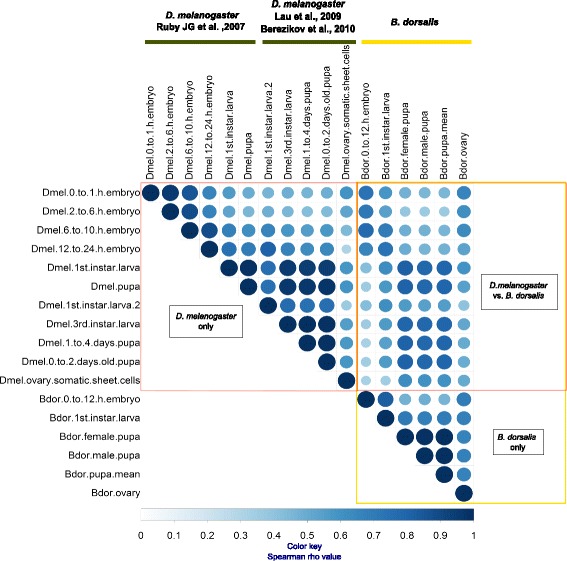
Correlation between miRNA abundance in *B. dorsalis* and *D. melanogaster* across developmental stages. Two publicly available *D. melanogaster* datasets were used to compare with the present study. Circle size and color represent correlation strength (spearman rho-value). All correlations were significant at p-value <0.01 (see Additional file 5: Table S5)

Reciprocal best BLAST hits between the hairpin precursors of the set of 109 initially identified *B. dorsalis* miRNAs, and those of the 256 known *D. melanogaster* miRNAs (as of miRBase relase#21), identified 68 presumed ortholog between both species, and these orthologus pairs were used to compare miRNA developmental expression profiles between both species. For that purpose, the *D. melanogaster* miRNA expression values across developmental stages were retrieved from the Gene Expression Omnibus (GEO) [28, 36] (GEO Accessions: GSM322208, GSM322219, GSM22245, GSM322338, GSM360256, GSM360257, GSM360260, GSM360262, GSM385744, GSM385748, GSM385821, and GSM385822). Data for both organisms was compared using counts per million reads (cpm). The Spearman correlation coefficients (ρ) between orthologous miRNA expression for all the life stages across both species ranged between 0.3 and 0.9, and all were highly significant, with p-values ranging between 0 and 0.01 (Additional file 5: Table S5 and Fig. 3). Strong correlation (ρ > 0.65) was observed between the *B. dorsalis* embryo library and the four embryo libraries from *D. melanogaster*, with the strongest correlation to the *D. melanogaster* 6 to 10 day embryo. This precise stage (6 to 10 days old embryo) in *D. melanogaster* is referred to as the ‘phylotypic’ stage, meaning that the most homologous developmental stage between different species occurs at this particular point [37]. Supporting this theory, expression of miRNAs in the *B. dorsalis* first instar library from [36], showed stronger correlation with this late *D. melanogaster* embryo than with first instar larva for this species. Pupae libraries from both species showed high correlation with other pupae libraries and larvae across the three studies, indicating that the population of miRNAs is more constant between these two stages. The ovary miRNA expression data from our *B. dorsalis* dataset correlated best with *D. melanogaster* eggs of up to 10 h. Correlation of the *B. dorsalis* ovary with *D. melanogaster* ovary somatic sheet cells was relatively poor (ρ = 0.5), this finding is not surprising since the *B. dorsalis* ovary library was composed of fertilized and fully developed ovaries, perhaps more reminiscent of just laid eggs, whereas the ovary somatic sheet cell (SSC) line represents only a fraction of the ovarioles.

**Fig. 4:**
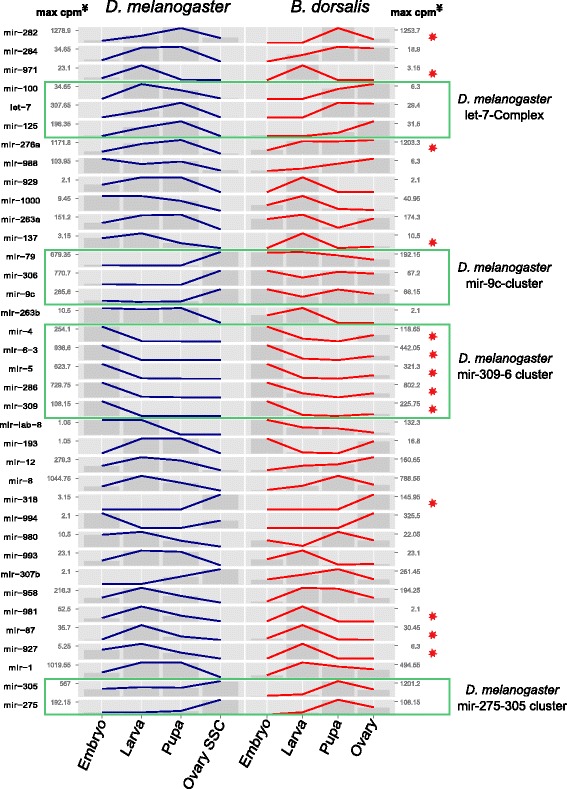
Trend plots of expression of statistically differentially regulated miRNAs in *D. melanogaster* and *B. dorsalis*. Plots were draw on a free scale to compare trends. Red asterisk show trends that are similar in both species. Green boxes show known miRNA clusters in *D. melanogaster* that were also found in this study. ^¥^Maximum count per million reads

To investigate whether the trend of expression of a particular miRNA in *B. dorsalis* was similar to that of the corresponding *D. melanogaster* orthologs, the 37 significantly differentially regulated *B. dorsalis* miRNAs side-by-side with their *D. melanogaster* counterparts, and a trend line of counts per million across the developmental stages on a free scale was generated (Fig. 4).

Visually inspecting each of the miRNA trend plots, there were 12 miRNAs with nearly identical expression patterns, and an additional miRNA (mir-276a), with a pattern varying only in the libraries derived from ovary tissue, which were the libraries with lowest correlation between the species. Among these twelve miRNAs with identical expression pattern, mir-282 was highly expressed in pupae of both *D. melanogaster* and *B. dorsalis* studies. Other studies have consistently found miR-282 to be expressed both in pupae and female adults of *D. melanogaster* [35, 38], and this miRNA has been shown to regulate viability and production of eggs through the targeting of the nervous-specific adenylate cyclase in pupae during metamorphosis [39]. Mir-276a, had an equivalent trend in expression from embryo to pupa; however, the high expression of this miRNA in *D. melanogaster* ovary SSC was not observed in the fertilized *B. dorsalis* ovary library. This miRNA also had highest expression in pupae in both species, and is also implicated in neural development, specifically in olfactory response [40]. Mir-137, mir-981, mir-87, and mir-927 had analogous expression patterns across both species, with highest expression observed in larval tissues, although their absolute expression was low compared to other significantly differentially regulated miRNAs. Even though these miRNAs were identified in genome-wide studies and computationally predicted in *D. melanogaster* [34, 36], we could only find one report of Dme-mir-87, showing its expression in relation to hormonal signaling [41], where mir-87 was found poorly expressed in early larva (in agreement with our data for *B. dorsalis* and the genome-wide studies in *D. melanogaster*), but then highly expressed in pupae. Finally, of the four conserved genomic miRNA clusters identified in *B. dorsalis*, only the mir-309-6 cluster (dme-mir-4, dme-mir-6-3, dme-mir-5, dme-mir-286, and dme-mir-309) had an analogous expression pattern across life stages.

### Genomic clusters of miRNAs in *B. dorsalis*

Clustered miRNA genes are fairly common in metazoan genomes. Approximately 40 % of described miRNA in nematodes, flies and mammals are localized in tandem clusters of two or more miRNAs less than 10 kb apart [27]. Ten genomic scaffolds with two or more of the 110 initially identified miRNAs were on the same strand, with a maximum distance of 3.5 kb between the most distant miRNAs in the largest cluster. An inspection of orthologs to these miRNA clusters in the *D. melanogaster* genome revealed that nine of these miRNA clusters are conserved in *D. melanogaster* (Table 4). The arrangement of miRNA genes in the cluster (order and direction) was the same in all *B. dorsalis* and *D. melanogaster* clusters except in cluster mir309-6, however, the *B. dorsalis* clusters appear to consistently span larger regions (of about 3 Kbp), compared to *D. melanogaster* (1kpb).

**Table 4:** miRNA clusters in *B. dorsalis*

miRNA	Scaffold	Coordinates	Strand	Mature sequence	Mature read count
bdo-mir-2a-2	scaffold00002	2204161..2204222	-	ucacagccagcuuugaugagcua	11192
bdo-mir-2a-1	scaffold00002	2204624..2204688	-	uaucacagccagcuuugaugagcu	7541
bdo-mir-2b	scaffold00002	2205153..2205237	-	uaucacagccagcuuugaggagcg	10527
bdo-mir-11593	scaffold00003	476312..476370	+	uccaugaaauucuguaauucug	1914
bdo-mir-11594	scaffold00003	477604..477663	+	guccaugaaauucuuuauuucug	224
bdo-mir-11	scaffold00005	2323679..2323748	+	caucacagucugaguucuugcu	2123
bdo-mir-998	scaffold00005	2326077..2326163	+	uagcaccaugagauucagcuc	243
bdo-mir-100	scaffold00010	2862114..2862173	+	aacccguaaauccgaacuugug	82
bdo-let-7	scaffold00010	2862318..2862376	+	ugagguaguagguuguauagu	516
bdo-mir-125	scaffold00010	2862662..2862722	+	ucccugagacccuaacuuguga	196
bdo-mir-2c	scaffold00020	1019361..1019426	+	ucacagccagcuuugaugagca	4392
bdo-mir-13b	scaffold00020	1021893..1021957	+	uaucacagccauuuugacgaguu	2289
bdo-mir-9b	scaffold00027	1320119..1320184	-	ucuuuggugauuuuagcuguaug	1582
bdo-mir-79	scaffold00027	1321251..1321313	-	uaaagcuagauuaccaaagcau	4037
bdo-mir-306	scaffold00027	1321793..1321859	-	ccagguacuuagugacucuca	1412
bdo-mir-9c	scaffold00027	1324096..1324160	-	ucuuugguauucuagcuguaga	1336
bdo-mir-5	scaffold00054	203234..203297	-	aaaggaacguucguugugauau	676
bdo-mir-4	scaffold00054	203558..203615	-	auaaagcuagacaaccauugca	668
bdo-mir-309b	scaffold00054	203736..203796	-	ucacuggguaaaguuuguccca	542
bdo-mir-6	scaffold00054	203902..203964	-	uaucacaguggcuguuccuuau	1764
bdo-mir-5b	scaffold00054	204040..204101	-	uaucacagugauuuuccuugu	953
bdo-mir-286	scaffold00054	204943..205010	-	ugacuagaccgaacacucgugcu	5017
bdo-mir-309	scaffold00054	205266..205336	-	ucacuggguaaaguuuguccu	1086
bdo-mir-5c	scaffold00054	206688..206749	-	uaucacagugauuuuccuugu	953
bdo-mir-12	scaffold00073	1266978..1267046	+	ugaguauuacaucagguacugg	1833
bdo-mir-304	scaffold00073	1266554..1266619	+	uaaucucaauuuguaacugugag	487
bdo-mir-283	scaffold00073	1264481..1264548	+	aaauaucagcugguaauucugg	339
bdo-mir-994	scaffold00147	463832..463903	-	uaaggaaauaguagccgugau	1180
bdo-mir-318	scaffold00147	463659..463730	-	ucacugggcuuuguuuaucuca	343
bdo-mir-305	scaffold03687	2585..2645	-	auuguacuucaucaggugcucugg	18881
bdo-mir-275	scaffold03687	2783..2848	-	ucagguaccugaaguagcgcgcg	1720

**Fig. 5:**
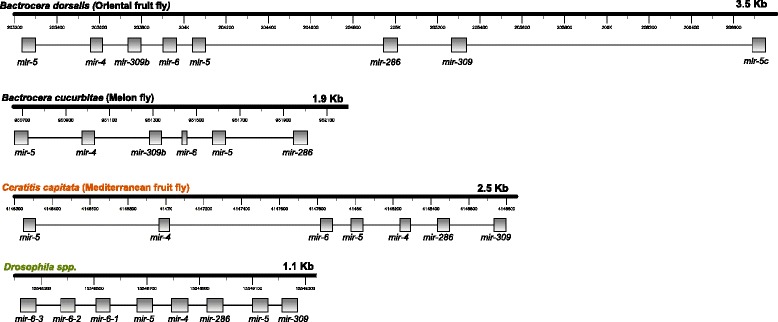
Comparison of the mir309-6 cluster across Drosophilids and three Tephritid fruit flies. Sequencing of small RNAs from *B. dorsalis*, identified six members of this cluster previously believed to be specific for Drosophilids. The search was expanded to other two Teprhitid species for which genome information is available (*Bactrocera cucurbitae*, melon fly; and *Ceratitis capitata,* mediterranean fruit fly) yielding similar clusters. The arrangement and spanning length of the miRNAs in the cluster in the Tephritid cluster seem to significantly differ from that in Drosophilids

The *D. melanogaster* mir-309-6, was previously reported as Drosophilid-specific [42, 43]. However, eight members of the mir-309-6 cluster were mapped in a region of 3.5 kb in Scaffold000054 from the *B. dorsalis* assembly. Although the general arrangement of the region differed significantly to that of *D. melanogaster*, a search of this sequence in the recently published *B. cucurbitae* (melon fly) genome assembly (NCBI accession GCA_000806345.1) revealed the same arrangement of miRNAs in *B. dorsalis*, except for the region where the two duplicated miRNAs were found. Moreover, comparison to the Mediterranean fruit fly (*Ceratitis capitata*) genome assembly (NCBI accession GCA_000347755.1), showed the presence of the same miRNAs in a different arrangement (Fig. 5).

The mir-309-6 cluster has been implicated in maternal transcript destabilization, the removal of transcripts maternally provided during oogenesis [44, 45]. Consistent with this observation, the expression of the mir-309-6 cluster was highest at the start of zygotic transcription in the early embryo in *D. melanogaster*. Transcripts for this in *D. melanogaster* were found subsequently depleted, but mature miRNAs could still be detected in larval stages [46, 47]. In our data for *B. dorsalis*, all miRNA members of this cluster were found to be very abundant in embryos, with counts per million reads comparable to those in *D. melanogaster*. While some miRNAs were still detected in the larval stage, no mature miRNAs were found in pupal samples, and a significant number of miRNAs were detected in fertilized ovary tissue (see next section). Taken together, this data indicates that this miRNA cluster is not specific to Drosophilids as previously believed, and that it has undergone extensive evolutionary divergence. Functionally, while the *D. melanogaster* mir-309-6 cluster was found to act as part of the zygotic machinery in the removal of maternal mRNAs, and is highly expressed in embryos, our *B. dorsalis* data indicates that the mir-309-6 cluster is functional before egg laying, either because zygotic transcription starts earlier than in *D. melanogaster*, or because these miRNAs are not only part of the zygotic machinery for maternal transcript decay, but also part of the maternal machinery, which plays the same role (removal of maternal mRNA) before the onset of zygotic transcription.

## Conclusions

Deep sequencing of small RNAs has allowed the identification of miRNAs present at four different life stages of the Tephritid fruit fly *Bactrocera dorsalis*. Sixty-nine miRNAs homologs to miRNAs in other species were identified with high confidence, and sufficient evidence was gathered to identify eleven additional miRNAs that were not previously reported. The latter may include conserved miRNAs with relatively low expression, and/or miRNAs that have evolved independently and are specific to the Tephritid family. The three replications per library allowed for a robust differential expression analysis of the identified miRNAs and their classification into life stage specific groups; miRNAs falling in these categories could be considered of importance because they are likely involved during transitional stages in development. To complement the data, and to provide more biological insight, we attempted to provide a list of potential targets for the identified miRNAs. Given that in metazoans, perfect complementarity to only six nucleotides in the seed region of the small RNA and the target is sufficient to promote RNA silencing, like in Drosophila, the resulting list of candidate mRNA targets was very extensive [21, 48], even with the stringent parameters we set for the miRANDA output. Although the dataset of potential targets was narrowed down to a short list of miRNAs for which RNAseq information was available, there is still need for wet lab experiments for confirmation of the targets.

Taking advantage of the vast resources and data available for the model species *D. melanogaster*, comparative analysis across conserved orthologous miRNAs were utilized to further support our findings. High correlation was identified between datasets at the level of abundance across developmental stages. Moreover, groups of miRNAs that are physically clustered in genomic regions were also found to be conserved between both *B. dorsalis* and *D. melanogaster*. Although these miRNA clusters differed in genomic spacing between the two organisms, this difference was consistent for all the clusters, being *B. dorsalis* clusters arranged in regions up to three times longer than *D. melanogaster* miRNAs but with the same order of the miRNAs in the cluster. Only one of the miRNA clusters, the mir309-6 showed poor conservation including repeated miRNAs and a different arrangement. Although genome assembly errors were a possibility, the same cluster, with the same arrangement was found in the sequenced genomes of the two other Tephritids, namely *B. cucurbitae*, and *C. capitata*. With the data available for *B. dorsalis*, we hypothesize that this cluster, which has highly diverged from the *D. melanogaster* dme-mir-309-6 cluster, may also function in maternal transcript destabilization machinery as it does in Drosophilids, however, because it is also expressed in ovaries, it may not be specific for the zygotic machinery. Importantly, this cluster was previously reported as being specific for drosophilids, and we proved that is not, demonstrating that this dataset, and similar datasets from Tephritids can be used as comparative tools for flies and other insects, to draw more robust conclusions about evolutionary questions.

Knowledge on miRNAs in *B. dorsalis* could help in developing novel pest control strategies, for example, miRNAs that are specific for egg and larval stages, likely involved in key pathways for developmental transitions, can be further characterized and utilized in miRNA mimics feeding and plant expression [48, 49]. Because miRNAs are very important in controlling developmental states, miRNA mimics targeting female specific sex determination and development transcripts could be used to generate genetic sexing strains that can be utilized in Sterile Insect Technique (SIT). Finally, this dataset could be further explored to find other specific regulatory pathways of interest, and as an aid for functional characterization of genes.

## Additional files

Additional file 1: Table S1. Full Rfam classification of sequenced small RNAs. All sequenced small RNAs were classified into RNA types according to the Rfam database. Diversity refers to the number of unique reads that fell into one of the RNA types. Frequency refers to number of different families found within each type. Abundance refers to the total number of reads found within each RNA type (XLSX 35 kb) Additional file 2: Table S2. 75 miRNAs with strong evidence of being known miRNAs in other species (XLSX 37 kb) Additional file 3: Table S3. Potential targets for the identified miRNAs. Annotated with Drosophila orthologs. (XLSX 321 kb) Additional file 4: Table S4. Differential expression in miRNAs between life stages. Fold changes and p-values of the 10 pairwise comparisons between samples. (XLSX 22 kb) Additional file 5: Table S5. Spearman's coefficients (ρ) and p-values for the correlation between expression values of *D. melanogaster* and *B. dorsalis* orthologous miRNAs across life stages. (XLSX 17 kb)
